# COVID-19 as a Mirror: Reflecting the Pandemic of Racism and the Historical Roots of Health Inequities

**DOI:** 10.3390/ijerph22020273

**Published:** 2025-02-13

**Authors:** Tiffany J. Grant

**Affiliations:** Research & Data Services, University of Cincinnati, Health Sciences Library, Cincinnati, OH 45267, USA; joffritm@ucmail.uc.edu; Tel.: +1-(513)-558-9153

**Keywords:** structural racism, health disparities, social determinants of health, medical mistrust, health equity, COVID

## Abstract

Historically, the attribution of biology to race has shaped societies and manifested in innumerable disparities and irreparable harm, especially in communities of color. From the earliest days of the United States to the present day, the dehumanization and “othering” of African Americans have caused deep racial inequities that have been perpetuated and embedded in American culture. The early months of the COVID-19 pandemic underscored the deep racial inequalities in the US, especially in health outcomes for communities of color. Structural racism has played a critical role in exacerbating disparities, with Black, Hispanic, Latinx, and Indigenous populations experiencing higher rates of severe disease and mortality. The interconnectedness of racism with the social determinants of health, concomitant with higher rates of chronic illnesses like diabetes and hypertension, increases vulnerability to severe COVID-19. Health disparities are compounded by implicit biases in the medical field, a lack of diversity among healthcare providers, and historical medical mistrust among marginalized groups. Underrepresentation in the medical field, biomedical sciences, and academia hinders efforts to address health disparities effectively. This essay seeks to raise awareness of how the concepts of race and racism have resulted in racial hierarchies that perpetuate systems of oppression and impede efforts toward racial and health equity. Specifically, this essay covers time periods in American history, including slavery, the Jim Crow Era, the Civil Rights Movement, and the COVID-19 pandemic, and discusses how addressing race and racism and the achievement of racial health equity require targeted efforts to increase diversity in healthcare and biomedical fields, improve cultural competence, and foster trust between medical professionals and communities of color.

## 1. Introduction

COVID-19 has presented irrefutable evidence of racial inequities in the United States, and these inequities, in concert with the COVID-19 pandemic, have converged to perpetuate the cycles of disparity that have profound and lasting impacts on communities of color. Throughout the pandemic, data analyses indicated that people of color experienced higher rates of disease and death than White people when data are adjusted to account for differences in age by race and ethnicity, [1] and structural racism has been identified as a factor in COVID-19-associated health disparities [2]. One study analyzing data from 2026 counties across the US found that counties with higher death rates also have a higher proportion of Black residents. This same study also showed that for every 1% increase in Black residents, there was a resulting 0.9% increase in deaths from COVID-19 [3]. Similarly, high death rates from COVID-19 have plagued Hispanic, Latinx, and Indigenous communities, and racism has been called out as a factor perpetuating these disparities [4,5].

Termed a synergistic epidemic, or syndemic, where “two categories of disease are interacting within specific populations”, COVID-19 connected with systems of oppression and biological and social factors to ravage communities of color [6]. The syndemic theory, first posited in 2010 by Singer, asserts that syndemics emerge disproportionately, and cluster among certain populations, particularly those made vulnerable by social conditions. A syndemic “involves a set of enmeshed and mutually enhancing health problems that, working together in a context of deleterious social and physical conditions that increase vulnerability, significantly affect the overall disease status of a population” [7]. The social determinants of health have played a major role in influencing the impacts of COVID-19. Healthy People 2030 has defined the SDoH as conditions in the social environment in which people are born, live, learn, work, and play that affect a wide range of health, functioning, and quality-of-life outcomes and risks [8]. The SDoH are divided into five categories, and these include economic stability, educational access and quality, healthcare access and quality, neighborhood and built environment, and social and community context. Studies have shown that the SDoH, including poverty, limited education, limited access to quality healthcare, overcrowded housing, food insecurity, and racism, significantly impact health outcomes and often exceed the influence of genetics or access to healthcare services [8]. Research has consistently linked these adverse SDoH with racial health inequities. For example, a study revealed 56 of the 500 largest cities in the US have large life expectancy gaps, where individuals can live 20 to 30 years longer than individuals who live only a few miles away. Those with lower expectancy are most frequently racially minoritized individuals who live in high-poverty, racially segregated areas [9]. Adverse SDoH contribute to the prevalence of chronic illnesses such as diabetes, asthma, and hypertension; consequently, it is not surprising that African Americans are 60% more likely to be diagnosed with diabetes, 30% more likely to be diagnosed with hypertension, and 30% more likely to die from heart disease than White individuals [10]. These illnesses are associated with oppressive and racist structural factors and have been implicated as major factors in disparities in the rates of severe disease and death from COVID-19 in these communities.

Health equity and equitable health systems help improve overall preparedness and the public health response to pandemics like COVID-19. However, it is difficult to envision achieving health equity across racial groups without first tackling the issue of racial equity, as racism is a driver of poor health outcomes [11,12]. McNeely states that “health equity, which references fairness and social justice, “exists only when people have an equal opportunity to be healthy” [13]. This “equal opportunity” does not exist for all people, as people of color face many barriers to quality healthcare due to poverty, including implicit and explicit bias from the medical community, and lack of trust in medical professionals. Although race is predominately a social factor, the inaccurate and egregious attachment of biological consequences to race has led to the mistreatment of people of color for centuries. A 2016 study reported that medical students and residents held “false and fantastical” beliefs about biological differences between Black and White Americans, resulting in a racial bias in pain perception, and these biases resulted in inadequate treatment recommendations for Black patients’ pain [14].

When there is a lack of race concordance between patient and physician, patients of color are more likely to experience microaggressions during interactions with their physician, causing damage to the relationship between patient and provider [15]. During medical encounters, perceived racial discrimination is most common for Black Americans and Native Americans, at 12.3% and 10.7%, respectively, while only 2.3% of White individuals reported similar perceptions [16]. A recent study identified epistemic injustice, or the discounting of knowledge and lived experiences regarding their bodies and health, as the main manifestation of racism in the healthcare setting. These experiences lead to isolation and were associated with the exacerbation of existing medical mistrust and poor patient–clinician communication [17]. Patients who experience discrimination from their doctors are less likely to trust them, causing delayed medical care, decreased compliance, and prolonged suffering. Additionally, medical mistrust, a consequence of systemic racism, has deep historical roots, is a key driver in the health status of communities of color, and was a key contributor to COVID-19 vaccine hesitancy during the COVID-19 pandemic [18].

Although COVID-19 case and death rates in the Black community decreased over time, the vulnerabilities of the community revealed during the early months of the pandemic forced the acknowledgment that racism and health disparities are intrinsically linked. Called out as a public crisis, racism has been implicated in racial health inequities that fueled disparities in COVID-19 case and death rates [19]. Although not an opportunistic infection in the classic sense, COVID-19 inexplicably behaves like one, taking full advantage of the vulnerabilities caused by racism, ravaging the bodies of individuals already battling chronic illnesses and environmental harms, and unleashing a more severe and deadlier course of disease. We must acknowledge the impact that racism has on health equity. While COVID-19 did not reveal racism, it did reveal the undeniable legacy of oppression, resulting in disparities and sweeping health inequities among communities of color that have been perpetuated over centuries. To ignore the existence of structural racism is to ignore the root cause of these disparities, thus perpetuating cycles of oppression, chronic disease, disparity, and premature death. Discussing racism is a way to bring awareness and to underscore its impact throughout society. Thus, this essay will explore the historical influence of racism in shaping our society, highlighting its profound and far-reaching negative effects on the health of people of color—effects that were brought into sharp focus during the COVID-19 pandemic. This essay will then issue a call to action and put forth suggestions for targeted efforts to increase diversity in the healthcare fields, improve cultural competence, and foster trust between doctors and patients to bring us closer to achieving health equity.

## 2. Methods

This study examined the historical influence of race and racism on US culture and the Black community, focusing on slavery, the Jim Crow Era, and the post-Civil Rights Movement as critical racialized time periods in US history. To assess the origins of race and racism, original documents, biographies of key figures, and relevant texts were identified and examined. Relevant information was extracted from digital databases, museum archives, news reports, and peer-reviewed articles. Search terms used to query Google Scholar and PubMed included the following: “racism AND health”, “racism AND health disparities”, “redlining”, “redlining AND social determinants of health”, “Jim Crow”, “Jim Crow AND health”, and “segregation AND health”. For COVID-19-related information, contemporary news reports and peer-reviewed articles were accessed via Google Scholar and PubMed. Search terms used included the following: “COVID-19 AND racial disparities”, “COVID-19 AND social determinants of health”, COVID-19 AND racism, and COVID-19 AND race. Searches were conducted July 2023 through December 2024, and relevant articles published between February 2010 and August 2024 were included. Articles and information that did not focus predominately on racism and the experiences of Black Americans were excluded. The author would like to acknowledge that this work was conducted by a sole author, which could introduce potential selection bias in reviewing and including papers for this study.

Although this essay focuses predominantly on African Americans, examining the impact of racism, slavery, Jim Crow, and segregation on health disparities in the Black community, the author acknowledges that other racial and ethnic groups in the US experience systemic racism and health inequities that were also exacerbated during the pandemic. Thus, other racial, ethnic groups are discussed in context throughout the text.

## 3. Results

### 3.1. Origins and Legacy of Scientific Racism and Racial Hierarchies

The earliest definitions of race came from 17th century French physician and philosopher, Francois Bernier, and the 18th century Swedish biologist, Carl Linneaus. Both used geographical location and physical appearance to make racial distinctions to divide humanity into distinct groups. Although Bernier did not rank the races, he notably described the European race as “the first race” and indicated that those within the race were within areas of “high civilization”, which included all of Europe. Also included in the first race were other areas of the world with populations similar in physical appearance to the Europeans, and his descriptions of the other races were described as deviations from the European “norm” [20]. In their descriptions of races, both Bernier and Linneaus asserted that the physical differences that separated races resulted from differences in biology between them. This correlation of race with biology was not only faulty but dangerous, as it provided false scientific justification for the belief that certain races were intrinsically superior or inferior, and this pseudoscientific link has supported and reinforced discriminatory practices and social hierarchies for centuries. The racial descriptions made by Bernier and Linneaus inevitably created a hierarchy within humanity, placing those of European descent at the top and all other races below, inadvertently paving the way for scientific racism, which has had centuries-long devasting and deadly consequences.

It must be noted that while Bernier and Linneaus established a racial classification system, their descriptions only reinforced the prevailing Eurocentric view that Europeans were superior. Claims of racial superiority by Europeans have consistently been used to justify the exploitation and mistreatment of people of color [21]. “For much of the period from the 15th century till now, during which Europeans and Africans have been connected through trade, empire and migration, both forced and voluntary, Europe has viewed the people of Africa through the distorting veil of racism and racial theory. In the British case much of the jumble of stereotypes, pseudo-science and wild conjecture that coalesced to form racism arose from the political battles fought over the slave trade and slavery, during the last decades of the 18th century and the first decades of the 19th” [21]. Racism, the biological need for self-preservation, and the desire for superiority invented hierarchical classifications of race that viewed those of African descent as subhuman.

The notion that Africans were less than Europeans justified numerous atrocities against the citizens and descendants of citizens of Africa, including, most importantly, the Atlantic Slave Trade, which removed Africans from Africa and transported them to other countries where they were forced into slavery. The dehumanization of Africans by European slave traders helped to justify slavery, particularly in the United States where the founding fathers had declared that all men were created equal [22]. Thus, slavery in the United States suggested that humanity ended at the shores of Africa, and European-descended American colonists were free to enslave Africans and those of African descent. In the United States, “race” became a mechanism for identifying non-White individuals, further distinguishing “races” of savages (Native Americans) and those deemed subhuman (people of African descent) [23].

Pseudoscientific theories of race helped to establish “legal categories based on the premise that Black and Native Americans were different, less than human, and innately, intellectually, and morally inferior—and therefore subordinate—to White individuals” [22]. These pseudoscientific theories, such as those provided by Samuel Cartwright, who pathologized enslaved people’s attempts to escape and attributed “laziness” among slaves to a disease, provided a medical justification for slavery. Cartwright, chairman of a Louisiana State Medical Association committee and respected doctor, notoriously promoted pseudoscientific and racist medical theories through his fabrication of diseases that explained resistance to slavery as pathological. Most infamous are his drapetomaia and dysaethesia aethiopica, which he claimed caused slaves to flee from captivity and laziness, respectively. Cartwright contended that enslaved people were created to be subservient and became mentally ill with either drapetomaia or dysaethesia aethiopica when they were allowed too much kindness or independence. Ironically, the punishment and cure for this disease was corporal punishment. Equally appalling is his assertion that the skin of Black Americans was thicker and less sensitive than White skin, and skin insensitivity was a symptom of dysaethesia aethiopica. Thus, to cure the disease, slaveowners were to lather the skin of the slave and whip them [24]. By framing resistance to enslavement as a mental illness, these theories reinforced the idea that enslaved people were inherently suited for servitude, and incapable of self-governance, thus legitimizing the institution of slavery for those inclined to adopt the practice. Cartwright’s explanations of his fabricated diseases reinforced the justification for slavery, and his false claims about the skin of Black individuals have had enduring harmful effects [24].

Although the Emancipation Proclamation outlawed slavery in the United States in 1963, oppression took on another form, with legalized discrimination brought on by the Jim Crow laws in the southern United States. The Jim Crow laws imposed extensive restrictions on the freedoms of Black Americans, limiting educational opportunities and economic advancement and prohibiting voting rights, reasserting the racial hierarchy and reaffirming White superiority. The laws institutionalized racial segregation, discrimination, and disenfranchisement, profoundly shaping the social, economic, and political lives of Black Americans. As early as the 1870s, states began to pass laws banning the integration of Black and White Americans [25]. In 1883, the US Supreme Court ruled that the Civil Rights Act of 1875, which was signed into law by then President Ulysses Grant and designed to guarantee Black Americans equal access to public accommodations, was unconstitutional. Through this landmark decision, the Court ruled that the 14th Amendment equal protection clause applied only to states and not to private citizens or businesses [25]. The impact of this ruling cannot be overstated, as it fundamentally altered the trajectory of civil rights in the United States and placed Black Americans in mortal danger. The legalization of discrimination had a devastating and lasting impact on Black Americans, as the Jim Crow laws reinforced stereotypes that dehumanized them and considered them unworthy of equality. These laws imposed systematic barriers that denied Black Americans access to quality education, healthcare, housing, and public infrastructure, creating and perpetuating cycles of poverty.

The legacy of Jim Crow shaped racial inequalities in wealth, education, criminal justice, and access to resources, and this continued for more than half a century until the Civil Rights Movement of the 1960s. Moreover, recent studies have shown that Black families make only 5 cents for every dollar obtained by White families [26]; Black Americans are incarcerated in state prisons at nearly five times the rate of White Americans; nationally, 1 in 81 Black adults are serving time in state prisons [27]; and Black students receive USD 3000 below the estimated adequate levels, indicating that disparities in wealth and education have been perpetuated over generations and are persistent today, “creating a self-sustaining cycle of unequal opportunity and unequal outcomes [28]”.

### 3.2. Racism, Redlining, and the Legacy of Inequality

The Civil Rights Act of 1964 effectively reversed the Jim Crow laws, but the impact of the racial segregation imposed by them had implications beyond the Civil Rights Movement. Discrimination against Black Americans during the Jim Crow era created systemic barriers to quality housing, employment, education and healthcare. Although the Civil Rights Act of 1964 granted access to previously forbidden resources and spaces, Black Americans still experienced disproportionately higher rates of poverty, a lack of education, diminished opportunities for employment, insufficient housing, and racism.

The Jim Crow era practice of redlining is one of the most pervasive examples of systemic racism that has led to generational disparities in access to housing, education, and healthcare. Redlining was the practice of financial lenders denying or limiting mortgage lending based on the neighborhood’s racial makeup. New, affluent, and racially homogenous neighborhoods were outlined in green, while Black and poor White neighborhoods were outlined in red. Those areas within the red lines were considered undesirable and unworthy of granting mortgage opportunities. The lines, whether green or red, were partly based on the belief that the presence of minoritized individuals would undermine property values and that areas with minoritized individuals were intrinsically unsafe [29]. Greenlined areas received investments for infrastructure, including schools, grocery stores, parks, access to quality healthcare, parks, recreational facilities, and public transportation, and their property values increased over time. Redlined areas went without such investments, resulting in deteriorating or lack of infrastructure, underfunded schools, lack of safe recreation facilities or spaces, increased and prolonged exposure to environmental hazards, food deserts, and limited or no access to quality healthcare. The lines ensured that areas would remain homogenous, guaranteeing that neighborhoods remained either White and rich, or poor and predominately Black or non-White [29,30].

Homeownership is a common mechanism to build generational wealth, as it allows families to build equity and pass down property that has appreciated in value over time [29]. The systematic exclusion of Black Americans from homeownership prevented this avenue of wealth building during the Jim Crow era, and this has had lasting implications. Decades after the Civil Rights Act of 1964 was passed, the generational impact of legacy and remnants of the Jim Crow laws and redlining remain evident, as individuals and communities of color still experience higher poverty rates, low(er) educational attainment, and live in low-income, disadvantaged neighborhoods [31]. The impact of the Jim Crow laws on the SDoH are still prevalent today, as data show that previously redlined areas are still predominately Black, and their inhabitants continue to face systemic barriers that lead to poorer health outcomes compared to White populations [29,32]. The segregation of Black Americans has resulted in the inability to acquire wealth and thus to find themselves on par with the socioeconomic status of the White majority in the US. Residential segregation and poverty are associated with many SDoH that result in poor health and poor health outcomes. Individuals living in poverty also contend with insufficient housing; environmental exposure to pollution and toxins; limited access to quality healthcare, employment and education; and an increased risk and rate of chronic diseases.

The historic discrimination and racism faced by Black Americans have manifested in poor health and health outcomes within the community, and the compounding nature of adverse SDoH has resulted in extensive health disparities that have critical health implications on the population [33]. The United States National Institute on Minority Health and Health Disparities has designated health disparity populations that are characterized by patterns of poorer health outcomes, indicated by the overall rate of disease incidence, prevalence, morbidity, mortality, or survival in the population as compared with the general population [34]. People of color and individuals with low socioeconomic statuses are counted in the population designation, indicating that Black Americans experience profound health disparities in comparison to Whites. A 2024 analysis that examined how people of color fared compared to White people across 64 measures of health, healthcare, and social determinants of health found that Black, Hispanic, and American Indian or Alaska Native people fare worse than White people across the majority of examined measures of health and healthcare and social determinants of health [35]. A review examining racial health disparities through the SDoH model found that people of color experience worse patient care and health outcomes in every clinical area studied [33]. In comparison to White Americans, Black Americans have a greater prevalence and earlier onset of chronic illnesses such as hypertension, arthritis, and cancer, and are twice as likely to develop diabetes or die from cardiac arrest [36], and while genetics may play a role, the environment and adverse SDoH are driving factors as well.

The environments shaped by poverty significantly impact health outcomes, creating conditions that increase the risk of chronic diseases. For example, residents of impoverished neighborhoods often lack access to affordable, healthy food options, creating food deserts that contribute to food insecurity and unhealthy dietary patterns [29]. These patterns increase the risk of obesity, a major risk factor for type II diabetes. Furthermore, environmental stressors such as pollution and the chronic stress associated with socioeconomic hardship contribute to an increased risk of hypertension, a critical risk factor for cardiovascular disease [37]. The combination of these chronic conditions—obesity, type II diabetes, and hypertension—exacerbated by systemic discrimination, creates a significant health burden within marginalized communities, rendering them particularly vulnerable during public health crises such as the COVID-19 pandemic. These disparities in chronic disease prevalence are not merely coincidental but are a direct consequence of the social and environmental conditions fostered by systemic inequities.

### 3.3. COVID-19: The Perfect Storm

Merriam Webster defines a perfect storm as a critical or disastrous situation created by a powerful concurrence of events [38]. The syndemic brought on by COVID-19, against the backdrop of systemic racism, economic inequities, and long-standing health disparities in the Black community, exposed and exacerbated the unequal burden of disease, lack of healthcare access, and social determinants of health that have historically plagued the Black community. COVID-19 has illuminated how racial inequities across multiple institutions in the United States have converged and unleashed a storm that has ravaged the country and decimated communities of color across the United States. The early impact of COVID-19 on communities of color has underscored how pre-existing social inequities amplified the impact of the pandemic and synergistically contributed to the higher infection rates, severe illness, and mortality seen in the Black community.

On 20 January 2020, the US confirmed its first case of COVID-19 from samples taken on 18 January 2020 in the state of Washington [39], and it soon became clear that there were racial and ethnic disparities in COVID-19 case and death rates. The staggering death rates of people of color from COVID-19 magnified their vulnerability, as they experienced case and death rates that were significantly higher than their percentage in the population [40]. The increased prevalence of hypertension, diabetes, and obesity in marginalized communities, concomitant with social and environmental conditions of overcrowded and multigenerational housing, jobs as essential workers preventing them from sheltering at home, and the need for public transportation, placed these communities at heightened risk for exposure to COVID-19 and worse outcomes if infected [41].

Numerous studies demonstrated how adverse SDoH significantly impacted the health outcomes of Black Americans early in the COVID-19 pandemic. A scoping review, assessing geographic inequalities in global COVID-19 mortality rates, found that 91% of the studies assessed demonstrated that COVID-19 mortality rates were higher in high-poverty areas in comparison to the rate in more affluent areas [42]. Spatial analysis assessing racial differences in COVID-19 disparities in the US found that areas with significant Black populations had higher income inequality than the national median, and areas with significant Black and Hispanic populations had a higher percentage of young people (<65 years) living without health insurance. These same areas were also correlated with high rates of COVID-19 [43]. Another study identified a significant association between the percentage of Black residents in a county and COVID-19 death rates, and this impact was dependent on the number of adverse SDoH in the county [3]. Researchers found that the number of healthcare providers in a racially advantaged zip code had an inverse impact on the number of COVID-19 cases, but racially disadvantaged zip codes had far fewer healthcare providers, further perpetuating the disparities in COVID-19 in these communities [2]. COVID-19 provided irrefutable evidence of the vulnerabilities of the Black community already suffering from high burdens of chronic illness brought on by racism and adverse social determinants of health. These findings emphasize the critical need for targeted interventions to address the root causes of health disparities and foster a more equitable mindset.

### 3.4. A Call to Action

Although disparities in cases and deaths have narrowed and widened over time, the underlying structural inequities in health and healthcare, and the social and economic factors that placed people of color at increased risk at the outset of the pandemic, remain. The COVID-19 pandemic and the social and racial unrest of the summer of 2020 have ushered in an era of awareness that is centered on diversity, representation, and equity and is aiming to reduce racism and its impact across multiple sectors. Achieving health equity is challenging when faced with racial and health disparities, but targeted interventions can aid in overcoming these barriers. Fostering efforts to increase diversity in the healthcare and biomedical fields is an essential step in the effort to decrease health disparities among people of color [44]. Evidence suggests that racial concordance between doctors and patients results in better population health, increased life expectancy, increased adherence to medication guidelines, improved communication, and increased screenings for chronic diseases [45,46,47,48]. These studies suggest that cultural similarities instigate more effective communication, trust, and confidence and mitigate implicit biases between doctors and patients.

Historically, people of color have been largely excluded from the medical and biomedical fields; thus, the numbers of Black physicians has increased by only 4% in the last 120 years [49]. After constant rejection from American institutions, James McCune Smith, the first Black American to practice medicine in the US, was forced to earn his medical, master’s, and baccalaureate degrees at Glasgow University in Scotland before returning to the US to practice medicine in 1837 [50]. Although the percentages of underrepresented healthcare workers are increasing, they remain well below their respective percentages in the general US population. Recent data from the American Association of Medical Colleges indicate that only 5.7% of US doctors identify as Black, while Black Americans make up 12% of the US population [51]. The same data show that Hispanics, Native Americans, and Native Hawaiians or Other Pacific Islanders constitute 6.9%, 0.3%, and 0.1% of the US physician workforce, respectively. A recent study indicated that although the healthcare population is becoming more diverse, people of color are found most often in entry-level or low-paying jobs [52]. Like the medical field, increasing diversity in the biomedical fields has also been problematic. Although individuals from diverse backgrounds earn 10% of biomedical science PhDs, they represent only 2% of the medical school and basic science teaching faculty [53]. The diversity of the biomedical workforce is only 13%, whereas the percentage in the general population is a combined total of 30%, indicating significant underrepresentation in these fields [54].

Academic institutions have a level of responsibility and accountability to the communities in which they reside; thus, there is a critical need for academic institutions to proactively implement efforts to recruit, retain, and train culturally competent biomedical professionals of color. In his presidential address to the Association for the Study of Higher Education, William Zumeta suggested that there is a social contract between higher education and the support of the public the institution is a part of, in that as the needs, values, and expectations of the public changes, the institution must adapt and respond accordingly [55]. The COVID-19 pandemic, concomitant with the death of George Floyed, prompted many academic institutions to reexamine their diversity efforts and incorporate more intentional efforts to promote diversity and inclusion in the education space. The academic response should be two-pronged, focusing both on recruitment and retention of students and faculty of color to foster inclusion and promote belonging to the biomedical fields. Where there is such a disparity between education, socioeconomic status, and health, universities have a social responsibility to provide opportunities that will level the field for those most impacted by these disparities in their own community. Recruitment strategies should start with schools in communities of color and extend to Minority-Serving Institutions, while retention efforts should emphasize training and education on facilitating inclusive and safe environments and providing mentoring to students and faculty of color. Institutions should set measurable goals for recruitment and retention, track progress, and refine the approach as needed.

Academic institutions are well positioned to improve the pipeline of the biomedical workforce into their communities through intentional efforts to provide early exposure of K-12 students to the sciences. Building early pipelines through collaboration between academic institutions and schools in predominately Black communities can help to foster a sense of belonging and science identity among students in these schools, preparing them for potential biomedical career opportunities. Summer research programs, internships, and mentorship programs that bridge the community and academic institutions provide excellent means of engagement and interaction. Many institutions already have biomedical summer research programs, and targeted recruitment through collaborations between K-12 schools and the institutions can help foster interest and increase success in biomedical fields. In addition, targeted and culturally competent recruitment campaigns that include materials designed to appeal to historically underrepresented groups and that highlight success stories and inclusive on-campus environments can help to stimulate genuine interest in higher education and help to bolster the pipeline between the community and the academic institution. Early engagement with science helps to demystify complex concepts and equips students with critical thinking and problem-solving skills, helping them overcome barriers and perform more confidently. However, while some of these may be low- or no-cost initiatives, other initiatives and programs will require investment from multiple sectors, including the government, nonprofit organizations, and others that specialize in developing curricula and have an equity-minded focus.

As investment in predominately Black communities has historically been minimal, it is essential for the health of these communities that investments in infrastructure prioritize equitable access to quality affordable housing, healthcare, and food. To ensure long-term sustainability and improve the overall health of these communities, community investment must focus on providing accessible healthcare and targeted programming to raise awareness of common health issues, such as diabetes and hypertension. Finally, any improvement in racial health outcomes must acknowledge the root cause of health inequities. None of what has been stated is new information, but there is a reluctance to acknowledge racism as the culprit for health inequities among communities of color. Instead, race is often used as a proxy for racism, and without context, many would believe that health inequities seen in the Black community and other communities of color are the fault of those in the community. There is danger in correlating race with biology, as this perpetuates harmful cycles of dehumanization, abuse, and exploitation facilitated by racism. Thus, intentional efforts to shift the focus from race to racism are necessary in the fight for both racial and health equity.

## 4. Conclusions

Achieving health equity requires an equity-focused mindset that is willing to acknowledge and call out racism as a root cause of health disparities in the Black community. We must acknowledge that while race creates divisions, there is no need for hierarchies among these divisions, as we are all human. To dismantle systemic racism and its impacts on health outcomes, we must begin to reframe our thinking on race and move forward with a more humanistic view that is inclusive and seeks more equitable outcomes for all races. This essay aims to catalyze conversations and actions to address health inequities and advocate for systemic change to build a more equitable society. The COVID-19 pandemic has resulted in tremendous suffering and loss across the globe, and while this is incredibly devasting, it would be even more so if we did not act on the knowledge gained.

## Data Availability

No new data were created or analyzed in this study. Data sharing is not applicable to this article.
